# The Price of Hospital Reshaping: Nasal Myiasis Caused by Flesh Fly (Diptera: Sarcophagidae) in Reallocated COVID-19 Intensive Care Unit

**DOI:** 10.3390/healthcare11111533

**Published:** 2023-05-24

**Authors:** Vladimir Dolinaj, Jasmina Grujić, Davor Križanović, Aleksandar Potkonjak, Thomas Pape, Pavle Banović

**Affiliations:** 1 Department of Anesthesia and Intensive Care, Clinical Centre of Vojvodina, 21000 Novi Sad, Serbia; krizasu@gmail.com; 2 Faculty of Medicine in Novi Sad, University of Novi Sad, 21000 Novi Sad, Serbia; jasmina.grujic@mf.uns.ac.rs; 3 Blood Transfusion Institute Vojvodina, 21000 Novi Sad, Serbia; 4 Department of Veterinary Medicine, Faculty of Agriculture, University of Novi Sad, 21000 Novi Sad, Serbia; aleksandar.potkonjak@polj.uns.ac.rs; 5 Natural History Museum of Denmark, Science Faculty, University of Copenhagen, 2100 Copenhagen, Denmark; tpape@snm.ku.dk; 6 Department of Microbiology with Parasitology and Immunology, Faculty of Medicine in Novi Sad, University of Novi Sad, 21000 Novi Sad, Serbia; 7 Department of Prevention of Rabies and Other Infectious Diseases, Pasteur Institute Novi Sad, 21000 Novi Sad, Serbia

**Keywords:** flesh fly, COVID-19, ICU, nasal myiasis, Serbia

## Abstract

Myiasis is a condition characterized by fly larvae infestation, most common in tropical regions, but with a risk of occurring anywhere in the world. Here, we report a case of nasal myiasis caused by a sarcophagid fly in a critically ill COVID-19 patient hospitalized in a reallocated ICU department in Serbia and discuss procedures that could prevent the occurrence of similar incidents in reallocated ICU departments worldwide.

## 1. Introduction

Since the severe acute respiratory syndrome coronavirus 2 (SARS-CoV-2) emerged in China in late 2019, the outbreak of the Coronavirus disease (COVID-19) very quickly grew into a pandemic that was officially declared by the World Health Organization (WHO) on March 11, 2020. Public health systems worldwide were unprepared to tackle the spread of SARS-CoV-2 [1,2], leading to an urgent need for hospitals to expand their capacities in order to withstand the large influx of critically ill COVID-19 patients and prevent a public crisis. 

Myiasis is a condition characterized by fly larvae infestation of the living body. It is most prevalent in tropical regions but may occur anywhere in the world. It is found most often in infants or in adult humans with deteriorated health, who are unable to protect themselves by keeping flies away or cleaning infected wounds. Myiasis is mainly detected in anatomic cavities, orifices and at sites with necrotic tissue, although some fly species (i.e., *Chrysomya bezziana*, *Cochliomyia hominivorax*, *Cordylobia anthropophaga*, *Dermatobia hominis*, and *Sarcophaga* spp.) can also invade healthy skin [3,4,5]. It was previously shown that parasitic infections account for 0.6–0.7% of intrahospital infections worldwide, while specific incidence and prevalence is dependent on the geographic region (around 1% in Western Europe) [6].

Here, we report a case of nasal myiasis caused by a sarcophagid fly in a critically ill COVID-19 patient hospitalized in a reallocated Intensive Care Unit (ICU) department in Serbia. 

## 2. Case Presentation 

A 65-year-old male patient was admitted to the COVID-19 department of the University Clinical Center of Vojvodina on 24 April 2021 with symptoms including signs of fever, shortness of breath, weakness, malaise, and nausea, and polymerase chain reaction (PCR) confirmed COVID-19. The patient had a background of rheumatoid arthritis, which was diagnosed four years earlier and was treated with prednisolone and methotrexate. He previously had several surgical procedures: total left hip arthroplasty, vocal cord polyp surgery, and septoplasty. The patient was not previously immunized against SARS-CoV-2. 

On the day of admission (seventh day of disease), the patient was without neurological deficit, febrile, spontaneously breathing with oxygen saturation (SpO2) 90% while breathing room air, tachycardic, hypotensive, and dehydrated. Chest x-ray confirmed progression of bilateral pneumonia (Supplementary Figure S1). Further clinical examination revealed limited mobility of knee joints and deformity of the fingers. Oxygen therapy via a face mask with flow rate 6 L/min was initiated as well as supportive medical treatment, according to the Serbian National COVID-19 Patient Treatment Guidelines. Antibiotic therapy was prescribed by an infectious disease specialist (Ceftriaxone and levofloxacin) due to elevated procalcitonin (PCT) level (Supplementary Table S1). On April 26 (third day of hospitalization; ninth day of disease), the patient’s general condition deteriorated, requiring increased oxygen support (flow rate 11 L/min). The deterioration of the patient was followed by an increased level of interleukin 6 (IL-6) (Supplementary Table S1), and a chest x-ray detected a progression of inflammatory changes in the lungs (Supplementary Figures S1 and S2). According to the Serbian National COVID-19 Patient Treatment Guidelines, anti–IL-6 receptor monoclonal antibody treatment (tocilizumab) was initiated, but without noticeable clinical improvement. 

On April 29 (6th day of hospitalization; 12th day of disease), the patient was transferred to a reallocated ICU due to advanced clinical deterioration manifested by elevated respiratory rate (35 respirations/min; normal range 14–20 respirations/min), increased needs for oxygen therapy (flow rate 15 L/min), hypoxemic respiratory failure, and progression of the inflammatory process in the lungs. 

Immediately after transfer to the reallocated ICU, oxygen therapy with high flow nasal cannula was initiated, with awake prone positioning and early respiratory rehabilitation treatment. Diagnostic and therapeutic procedures were performed according to the clinical condition and Serbian National COVID-19 Patient Treatment Guidelines. Five days later, respiratory failure advanced, and the patient was intubated in order for mechanical lung ventilation to be initiated. 

**Figure 1:**
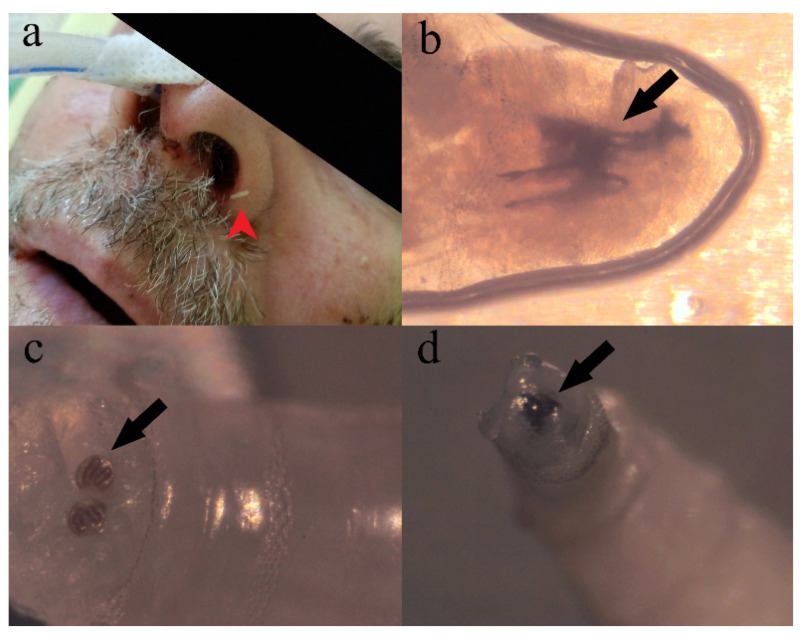
Case of infestation and *Sarcophaga* sp. morphology. (**a**) Patient on 17th day of hospitalization, red arrowhead pointing at *Sarcophaga* sp. first instar larva—note the presence of nasogastric tube in right nasal cavity; (**b**) *Sarcophaga* sp. first instar larva, wet mount, black arrow pointing at cephaloskeleton, lateral view; (**c**) *Sarcophaga* sp. first instar larva, posterior spiracles, black arrow pointing at kidney-shaped slits of posterior spiracles partially encircled by incomplete peritreme; (**d**) anterior end of the *Sarcophaga* sp. first instar larva, black arrow pointing at two oral hooks.

On May 9 (17th day of hospitalization; 22nd day of disease; 11th day in ICU), numerous dipterous larvae (approximately 20 larvae) were detected in the left nostril of the patient (Figure 1a). The larvae were removed manually, placed in 70% ethanol, and forwarded to Pasteur Institute Novi Sad for entomological examination. According to morphological keys [7], the samples were identified as the first instar larva of *Sarcophaga* sp. (Diptera: Sarcophagidae) (Figure 1b–d). After removal of all larvae, rhinoscopy showed normally colored mucosa and septum deviated to the left, due to presence of the nasogastric tube. Computerized tomography was indicated but not performed due to the hemodynamical instability of the patient, since vasopressor support was previously initiated. On May 12 (20th day of hospitalization; 25th day of disease; 14th day in ICU), treatment of the patient was further complicated with the development of sepsis and multi-organ failure (Supplementary Figure S2), and fatal outcome was reached one day later.

## 3. Discussion

Since the emergence of COVID-19 in early December 2019, a number of patients with severe and critical forms of the disease, which required treatment in an ICU, increased dramatically [8], outpacing hospital capacities and leading to a global public health crisis [9]. ICU resources were overwhelmed in many countries worldwide, including China [10], Italy [11], and Singapore [12]. Therefore, healthcare decision-makers became aware that an integral part of any pandemic response is to prepare ICUs with increased capacities in terms of both the number of beds and trained staff members [12]. Goh et al. gave practical considerations and strategies in preparing ICUs for the COVID-19 pandemic [12]. The main targets to be achieved were as follows: (i) preparation and implementation of rapid identification of COVID-19 cases and strict isolation protocols; (ii) provision of a sustainable workforce with a focus on infection control, (iii) assurance of adequate supplies to equip ICUs and protect healthcare workers; and (iv) maintenance of quality clinical management as well as effective communication [12]. Our institution is the largest academic tertiary medical center in the Serbian Northern Autonomous Province of Vojvodina, which has around 1,800,000 inhabitants. Since our institution was designed as a pavilion hospital, it was difficult to carry out the expansion of the ICU capacity during the COVID-19 pandemic since not a single building meets the requirements for the treatment of patients with infectious diseases.

Expansion of hospital capacities can be achieved via the construction of new healthcare facilities or the reorganization of existing hospital departments or public venues, which are repurposed and equipped for support of COVID-19 patients [13,14]. Reasonably low and middle-income countries, as well as some high-income countries, are more oriented toward the second option, i.e., the conversion of existing postoperative wards and other departments into improvised ICUs [14,15,16,17,18,19]. 

As in many hospitals worldwide, the existing bed capacities in the ICU at the Infectious Disease Clinic (Clinical center of Vojvodina) were not sufficient to admit a large number of patients requiring non-invasive and invasive mechanical ventilation, as well as other measures of intensive treatment and monitoring. Initially, surgical critical care units were transformed into ICUs, and when these facilities were outpaced, ICUs were reallocated to hospital wards, which, in the construction and technical sense, do not fulfill required standards, including the possibility of opening a window. 

Despite many advantages of hospital reshaping approaches, such as the preexisting infrastructure for water, food, electricity, and oxygen supply, newly formed COVID-19 ICUs are facing problems such as shortages of trained staff due to increased workload [16] and insufficient department construction if the facility is not designed to support critically ill patients. As a consequence of new organizational plan implementation and the rapid integration of new staff members, hygiene conditions may deteriorate and the emergence of unexpected nosocomial infections or infestations in reallocated COVID-19 ICU is possible, influencing the recovery of the critically ill patients [18].

According to the Centers for Disease Control and Prevention, ICUs for the COVID-19 critically ill should ideally consist of negative pressure airborne infection isolation rooms [20]; these are single-occupancy patient-care rooms used to isolate persons with a suspected or confirmed airborne infectious disease, with at least 6–12 air changes per hour, and with any recycled air filtered before recirculation [21,22]. Therefore, the transmission of infectious agents by droplet nuclei associated with coughing or the aerosolization of contaminated fluids is minimized [23]. Unfortunately, negative pressure airborne infection isolation rooms were not available in our COVID-19 ICUs. Consequently, we had to cohort patients in the buildings, which are not designed to contain ICUs. In order to prevent transmission of the infection to medical staff, ICU beds were two meters apart. Doctors and nurses were wearing personal protective equipment: face shield, N95 respirators, gloves, isolation gown, and shoe protection. The only possibility to obtain air circulation in the reallocated ICU was by opening the windows. This entailed additional risks such as the entry of insects into the ICU. 

As an academic tertiary medical center, we could not suspend all elective surgeries and non-essential services. A suspension would free up a certain number of beds in the ICUs, but this would endanger the lives of patients who did not suffer from COVID-19 and who needed medical services that could, in our country, only be provided in a tertiary medical center (e.g., neurosurgery, vascular surgery, and treatment of malignant diseases in ENT, etc.). In addition, we had to reserve a certain number of beds in our ICUs for non-COVID-19 emergency patients (e.g., polytraumatized cases, acute bleeding cases, etc.).

Infestation of nasal cavities by dipterous larvae is commonly reported from persons with deteriorated health living in tropical and subtropical regions [24,25,26]. Several cases of nasal nosocomial myiasis have been reported in other parts of the world [26,27,28,29], but to our knowledge, this is the first case from an ICU in Serbia. 

So far, nosocomial myiasis cases have been caused by several fly species, including *Lucilia sericata* [30,31], *Lucilia cuprina* [32], *Chrysomya bezziana* [33], and *Megaselia scalaris* [34], as well as by several species of *Sarcophaga* [35,36,37,38,39,40], among others. In addition to the development of the larvae (maggots) in patient tissues, contact of immunocompromised/ICU patients with flies introduces the risk of insect-borne pathogen transmission, leading to possible nosocomial bacterial superinfections [4]. 

Unlike the majority of cases of nosocomial myiasis reported previously [41,42,43,44], the nasal cavity of the present patient had normally colored mucosa without observable necrotic lesions. This is in accordance with the known life cycle of *Sarcophaga* spp., where females deposit motile first instar larvae (i.e., larviparous flies) [45], and we take this as evidence of an early infection with insufficient time for any tissue damage. Unfortunately, due to the patient’s hemodynamic instability, we were not able to perform computerized tomography and determine if there were lesions in the sinus cavities.

The risk factors for acquiring myiasis were identified as (i) poor hygiene and low socioeconomic status; (ii) an abundance of exposed preexisting suppurative lesions; (iii) immunodeficiency; and (iv) influence of climatic conditions, with maggots thriving under high humidity and warm weather. For nasal myiasis in particular, older age (i.e., >50 years of life) and poor nutritional status are considered as additional risk factors [46].

The most important risk factors for the occurrence of nosocomial myiasis are identified as the deliberation of blood or odors of decomposition, sedation and mechanical ventilation, poor nursing care, summer time, and numerous comorbidities that compromise the vascular status of immunosuppressed patients [43,47]. In addition, patients with draining, chronic wounds, and dressings soiled by purulent, mucoid drainage, or body fluids are also under higher risk for acquiring myiasis [48].

Several factors that could individually or concomitantly increase patient susceptibility for infestation by necrophagous fly larvae were identified in this study, and include (i) severe COVID-19 requiring mechanical lung ventilation and sedation, thus disabling the patient from keeping the flies away; (ii) immunosuppression due to prednisolone and methotrexate treatment combined with Tocilizumab leading to the weakening of patient immune defense mechanisms; and (iii) hospitalization in medical facility that was not originally planned to support the need of ICUs. Here, the patient fulfilled several risk factors; he was 65 years old with comorbidities (rheumatoid arthritis) and chronic immunosuppressive therapy. Furthermore, during his treatment, he received tocilizumab, a biological medication that affects the immune system by blocking the inflammatory protein IL-6. 

Untreated nasal myiasis may lead to septal or palatal perforation, orbital and facial cellulitis, and ulceration of the tonsils and posterior pharyngeal wall, leading to the extension of the infestation beyond its original source [49]. Therefore, early diagnosis and treatment are extremely important for the prevention of complications and the implementation of preventive measures.

According to White and colleagues, there is no consensus regarding a treatment standard for nasal myiasis. Nasal myiasis can be treated by nasal irrigation with weak solutions of chloroform, nasal packing with chloroform and turpentine, and the manual removal of maggots. Medical treatment by the usage of ivermectin or other anthelmintics in conjunction with endoscopic removal and saline irrigation is recommended [49]. Our intervention consisted of replacement of the nasogastric tube to another nostril before CT diagnostic was performed, since endoscopic diagnostic of the nasal cavity and removal were not technically feasible in the conditions of a reallocated COVID-19 ICU.

Regarding the prevention of nosocomial myiasis, Sherman emphasizes the necessity of efforts on two fronts: making the host less attractive (decreasing patient risk factors); and reducing fly populations in the healthcare environment, which is not an easy task. Since myiasis-causing flies are attracted to and induced to deposit eggs or larvae in response to the odors of necrotic organic matter [48], it is necessary for healthcare staff to pay attention to the oral and nasal hygiene of patients and to control cleaning frequency, room hygiene, and the proper handling and disposal of waste [48].

In addition, this incident highlights the importance of the One Health approach in the management of zoonotic diseases, as well as in the design of facilities that are repurposed for the hospitalization of patients requiring critical care. In that context, fly larvae infestations such as the one described here should not be the only concern for hospital management and healthcare practitioners; insect infestations of hospital environments directly from the urban surrounding have been documented in Europe and North America. The most important are cockroaches (Order Blattodea), poultry red mite (*Dermanyssus gallinae*) [50,51], and pharaoh ants (*Monomorium pharaonis*), which were introduced to Europe from tropical regions and have the ability to thrive indoors in temperate regions given adequate temperature and humidity [52]. In these cases, medical entomologists should be able to provide crucial information in relation to insects of medical and public health importance, conduct surveillance and data analysis, and communicate awareness of nosocomial infestations and arthropod-borne diseases to hospital management and healthcare practitioners. However, if the medical facility cannot include medical entomologist in its working teams, a consultation with entomologists, e.g., from a local University, should provide valuable insights that can allow decision makers to decide in which way hospital reshaping should be designed such that minimal risk for nosocomial infestation of this kind can occur.

## 4. Conclusions

To the best of our knowledge, this is the first reported case of nosocomial myiasis in a patient with COVID-19 hospitalized in a reallocated ICU. In order to achieve an environment for the best possible outcome of critically ill patients, it is necessary to develop awareness about nosocomial myiasis within medical professionals, especially when a reorganization of the health system is required during emergencies such as pandemics, floods, and earthquakes. Measures that could mitigate the risk of fly larvae infestation in reallocated ICUs are regular disinfection, the placing of mosquito netting on the windows, and the integration of expert entomologists into the team in charge of reallocation planning.

## Data Availability

Data sharing not applicable.

## Supplementary Materials

The following supporting information can be downloaded at: https://www.mdpi.com/article/10.3390/healthcare11111533/s1, Figure S1: Chest X-ray findings; Figure S2: Dynamics of pro-inflammatory markers during the course of hospitalization; Table S1: Laboratory findings in patient during the course of hospitalization.

## References

[1] Elhadi M., Msherghi A., Alkeelani M., Alsuyihili A., Khaled A., Buzreg A., Boughididah T., Abukhashem M., Alhashimi A., Khel S. (2020). Concerns for Low-Resource Countries, with under-Prepared Intensive Care Units, Facing the COVID-19 Pandemic. Infect. Dis. Health.

[2] Frutos R., Gavotte L., Serra-Cobo J., Chen T., Devaux C. (2021). COVID-19 and Emerging Infectious Diseases: The Society Is Still Unprepared for the next Pandemic. Environ. Res..

[3] Dutto M., Bertero M. (2011). Cutaneous Superficial Myiasis: Report of a Rare Nosocomial Parasitic Disease Caused by *Sarcophaga* spp. (Diptera, Sarcophagidae). Cent. Eur. J. Public Health.

[4] Fürnkranz U., Walochnik J. (2021). Nosocomial Infections: Do Not Forget the Parasites!. Pathogens.

[5] Hall M.R.J., Farkas R., Papp L., Darvas B. (2000). Traumatic Myiasis of Humans and Animals. Contributions to a Manual of Palaearctic Diptera, General and Applied Dipterology.

[6] Vincent J.-L., Rello J., Marshall J., Silva E., Anzueto A., Martin C.D., Moreno R., Lipman J., Gomersall C., Sakr Y. (2009). International Study of the Prevalence and Outcomes of Infection in Intensive Care Units. JAMA.

[7] Dodge H.R. (1953). Diptera: Pictorial Key to Principal Families of Public Health Importance. Pictorial Keys to Arthropods, Reptiles, Birds, and Mammals of Public Health Significance.

[8] Phua J., Weng L., Ling L., Egi M., Lim C.-M., Divatia J.V., Shrestha B.R., Arabi Y.M., Ng J., Gomersall C.D. (2020). Intensive Care Management of Coronavirus Disease 2019 (COVID-19): Challenges and Recommendations. Lancet Respir. Med..

[9] Fagiuoli S., Lorini F.L., Remuzzi G. (2020). COVID-19 Bergamo Hospital Crisis Unit. Adaptations and Lessons in the Province of Bergamo. N. Engl. J. Med..

[10] Zhao S., Sha T., Xue Y., Chen H. (2023). Flattening the Curve: Imperative When China Eases the Severe COVID-19 Control Policy. J. Infect..

[11] Grasselli G., Pesenti A., Cecconi M. (2020). Critical Care Utilization for the COVID-19 Outbreak in Lombardy, Italy: Early Experience and Forecast During an Emergency Response. JAMA.

[12] Goh K.J., Wong J., Tien J.-C.C., Ng S.Y., Wen S.D., Phua G.C., Leong C.K.-L. (2020). Preparing Your Intensive Care Unit for the COVID-19 Pandemic: Practical Considerations and Strategies. Crit. Care.

[13] Dondorp A.M., Papali A.C., Schultz M.J. (2021). Recommendations for the Management of COVID-19 in Low- and Middle-Income Countries. Am. J. Trop. Med. Hyg..

[14] Haldane V., De Foo C., Abdalla S.M., Jung A.-S., Tan M., Wu S., Chua A., Verma M., Shrestha P., Singh S. (2021). Health Systems Resilience in Managing the COVID-19 Pandemic: Lessons from 28 Countries. Nat. Med..

[15] Condes E., Arribas J.R., COVID-19 MADRID-S.P.P.M. Group (2021). Impact of COVID-19 on Madrid Hospital System. Enferm. Infecc. Microbiol. Clin. Engl. Ed..

[16] Lu X., Xu S. (2020). Intensive Care for Severe Acute Respiratory Syndrome Coronavirus 2 (SARS-CoV-2) in a Makeshift ICU in Wuhan. Crit. Care.

[17] Remuzzi A., Remuzzi G. (2020). COVID-19 and Italy: What Next?. Lancet.

[18] Shrestha G.S., Lamsal R., Tiwari P., Acharya S.P. (2021). Anesthesiology and Critical Care Response to COVID-19 in Resource-Limited Settings: Experiences from Nepal. Anesthesiol. Clin..

[19] Tempe D.K., Khilnani G.C., Passey J.C., Sherwal B.L. (2020). Challenges in Preparing and Managing the Critical Care Services for a Large Urban Area During COVID-19 Outbreak: Perspective from Delhi. J. Cardiothorac. Vasc. Anesth..

[20] Isolation Precautions Guidelines Library. Infection Control. CDC. https://www.cdc.gov/infectioncontrol/guidelines/isolation/index.html.

[21] Lee J.K., Jeong H.W. (2020). Rapid Expansion of Temporary, Reliable Airborne-Infection Isolation Rooms with Negative Air Machines for Critical COVID-19 Patients. Am. J. Infect. Control.

[22] CDC Healthcare Workers. https://www.cdc.gov/coronavirus/2019-ncov/hcp/infection-control.html.

[23] Mohapatra R.K., Pintilie L., Kandi V., Sarangi A.K., Das D., Sahu R., Perekhoda L. (2020). The Recent Challenges of Highly Contagious COVID-19, Causing Respiratory Infections: Symptoms, Diagnosis, Transmission, Possible Vaccines, Animal Models, and Immunotherapy. Chem. Biol. Drug Des..

[24] Caumes E., Carrière J., Guermonprez G., Bricaire F., Danis M., Gentilini M. (1995). Dermatoses Associated with Travel to Tropical Countries: A Prospective Study of the Diagnosis and Management of 269 Patients Presenting to a Tropical Disease Unit. Clin. Infect. Dis..

[25] Rana A., Sharma R., Sharma V., Mehrotra A., Singh R. (2020). Otorhinolaryngological Myiasis: The Problem and Its Presentations in the Weak and Forgotten. GMJ.

[26] Singh A., Singh Z. (2015). Incidence of Myiasis among Humans—A Review. Parasitol. Res..

[27] El Haj Chehade A., Metcalf J., Jacobs B. (2022). Nasal Myiasis under Direct Bronchoscopic Visualization. Respirol. Case Rep..

[28] Giangaspero A., Barlaam A., Pane S., Marchili M.R., Muda A.O., Putignani L., Hall M.J.R. (2021). Accidental Nasal Myiasis Caused by *Megaselia rufipes* (Diptera: Phoridae) in a Child. J. Med. Entomol..

[29] Kuo H., Meng X., Huang X., Wu A., Long L. (2021). Nasal Myiasis in Patients with Disturbance of Consciousness: A Case Report and Literature Review. Zhong Nan Da Xue Xue Bao Yi Xue Ban.

[30] Jang M., Ryu S.-M., Kwon S.-C., Ha J.-O., Kim Y.-H., Kim D.-H., Jung S.-M., Lee S.-I., Sohn W.-M., Cha H.-J. (2013). A Case of Oral Myiasis Caused by *Lucilia sericata* (Diptera: Calliphoridae) in Korea. Korean J. Parasitol..

[31] Kalezic T., Stojkovic M., Vukovic I., Spasic R., Andjelkovic M., Stanojlovic S., Bozic M., Dzamic A. (2014). Human External Ophthalmomyiasis Caused by *Lucilia sericata* Meigen (Diptera: Calliphoridae)—A Green Bottle Fly. J. Infect. Dev. Ctries..

[32] Nazni W.A., Jeffery J., Lee H.L., Lailatul A.M.N., Chew W.K., Heo C.C., Sadiyah I., Khairul A.M., Heah S.K., Mohd H.H. (2011). Nosocomial Nasal Myiasis in an Intensive Care Unit. Malays. J. Pathol..

[33] Mircheraghi S.F., Mircheraghi S.F., Ramezani H., Riabi A., Parsapour A. (2016). Nasal Nosocomial Myiasis Infection Caused by *Chrysomya bezziana* (Diptera: Calliphoridae) Following the Septicemia: A Case Report. Iran J. Parasitol..

[34] Wakid M.H. (2008). A Laboratory-Based Study for First Documented Case of Urinary Myiasis Caused by Larvae of *Megaselia scalaris* (Diptera: Phoridae) in Saudi Arabia. Korean J. Parasitol..

[35] Alizadeh M., Mowlavi G., Kargar F., Nateghpour M., Akbarzadeh K., Hajenorouzali-Tehrani M. (2014). A Review of Myiasis in Iran and a New Nosocomial Case from Tehran, Iran. J. Arthropod Borne Dis..

[36] Chigusa Y., Kawakami K., Shimada M., Kurahashi H., Matsuda H. (2006). Hospital-Acquired Oral Myiasis Due to *Boettcherisca septentrionalis* (Diptera: Sarcophagidae) in Shimane Prefecture, Japan. Med. Entomol. Zool..

[37] Dutto M., Bertero M. (2010). Traumatic Myiasis from *Sarcophaga* (*Bercaea*) *cruentata* Meigen, 1826 (Diptera, Sarcophagidae) in a Hospital Environment: Reporting of a Clinical Case Following Polytrauma. J. Prev. Med. Hyg..

[38] Giangaspero A., Marangi M., Balotta A., Venturelli C., Szpila K., Di Palma A. (2017). Wound Myiasis Caused by *Sarcophaga* (*Liopygia*) *argyrostoma* (Robineau-Desvoidy) (Diptera: Sarcophagidae): Additional Evidences of the Morphological Identification Dilemma and Molecular Investigation. Sci. World J..

[39] Suwannayod S., Sanit S., Sukontason K., Sukontason K.L. (2013). *Parasarcophaga* (*Liopygia*) *ruficornis* (Diptera: Sarcophagidae): A Flesh Fly Species of Medical Importance. Trop. Biomed..

[40] Uni S., Shinonaga S., Nishio Y., Fukunaga A., Iseki M., Okamoto T., Ueda N., Miki T. (1999). Ophthalmomyiasis Caused by *Sarcophaga crassipalpis* (Diptera: Sarcophagidae) in a Hospital Patient. J. Med. Entomol..

[41] Serafim R.A., do Espírito Santo R.B., de Mello R.A.F., Collin S.M., Deps P.D. (2020). Case Report: Nasal Myiasis in an Elderly Patient with Atrophic Rhinitis and Facial Sequelae of Leprosy. Am. J. Trop. Med. Hyg..

[42] Surayya R., Parwati D.R. (2021). Management of Nasal Myiasis and Type 2 Diabetes Mellitus: A Rare Case and Review Article. Int. J. Surg. Case Rep..

[43] Szakacs T.A., MacPherson P., Sinclair B.J., Gill B.D., McCarthy A.E. (2007). Nosocomial Myiasis in a Canadian Intensive Care Unit. CMAJ.

[44] Teah M.K., Chu Y.M., Shanmuganathan S.D., Yeap T.B. (2020). Massive Airway Myiasis: An Extreme Rarity. BMJ Case Rep..

[45] Hall R.D., Gerhardt R.R., Mullen G., Durden L. (2002). Flies (Diptera). Medical and Veterinary Entomology.

[46] Francesconi F., Lupi O. (2012). Myiasis. Clin. Microbiol. Rev..

[47] Barazi R., Dabbous H. (2020). Nasal Myiasis in Pediatric Age Group: Case Report and Review of the Literature. Ann. Clin. Otolaryngol..

[48] Sherman R.A., Roselle G., Bills C., Danko L.H., Eldridge N. (2005). Healthcare-Associated Myiasis: Prevention and Intervention. Infect. Control Hosp. Epidemiol..

[49] White Z.L., Chu M.W., Hood R.J. (2015). Nasal Myiasis: A Case Report. Ear Nose Throat J..

[50] Barlaam A., Puccini A., Caiaffa M.F., Di Bona D., Macchia L., Giangaspero A. (2022). Dermanyssosis in the Urban Context: When the One Health Paradigm Is Put into Practice. Pathogens.

[51] Bellanger A.P., Bories C., Foulet F., Bretagne S., Botterel F. (2008). Nosocomial Dermatitis Caused by *Dermanyssus gallinae*. Infect. Control Hosp. Epidemiol..

[52] Flamm H., Rotter M. (1999). Angewandte Hygiene im Krankenhaus und Arztpraxis.

