# Supplementary figures and images for: The Price of Hospital Reshaping: Nasal Myiasis Caused by Flesh Fly (Diptera: Sarcophagidae) in Reallocated COVID-19 Intensive Care Unit
Source: Healthcare (Basel). 2023 May 24;11(11):1533. doi: 10.3390/healthcare11111533 (PMC10252984; doi:10.3390/healthcare11111533)

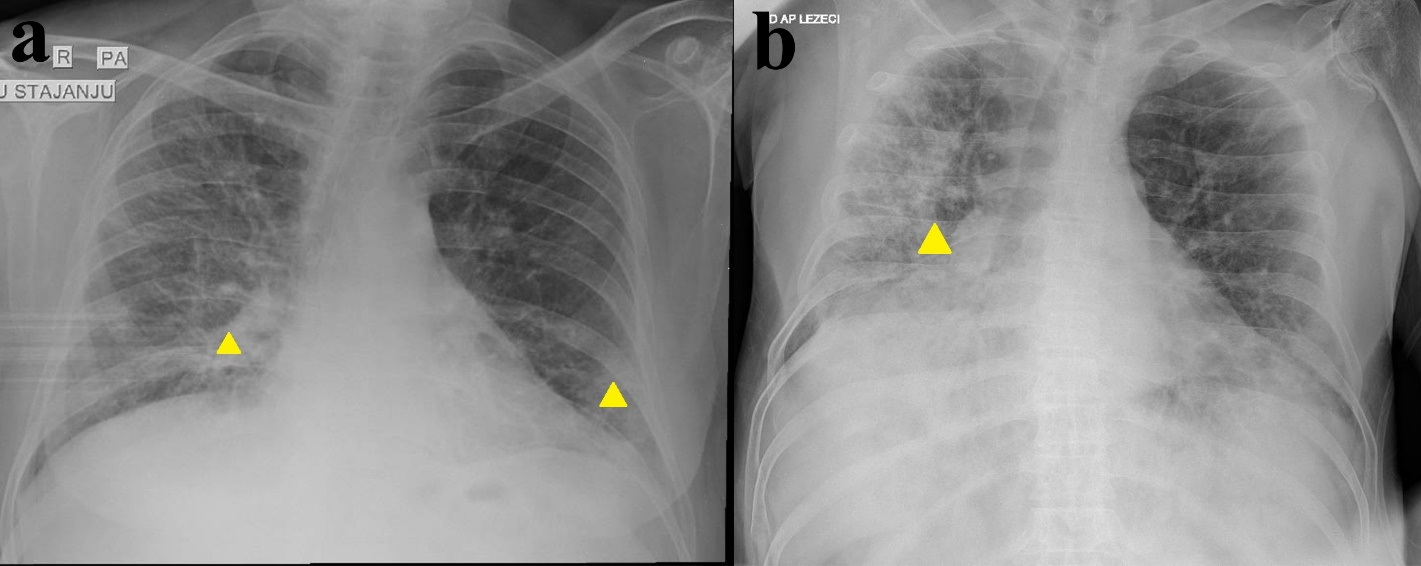

Supplement: Supplementary file 1 [file healthcare-11-01533-s001.zip › Supplementary figure S1 Chest X-ray findings.tif]

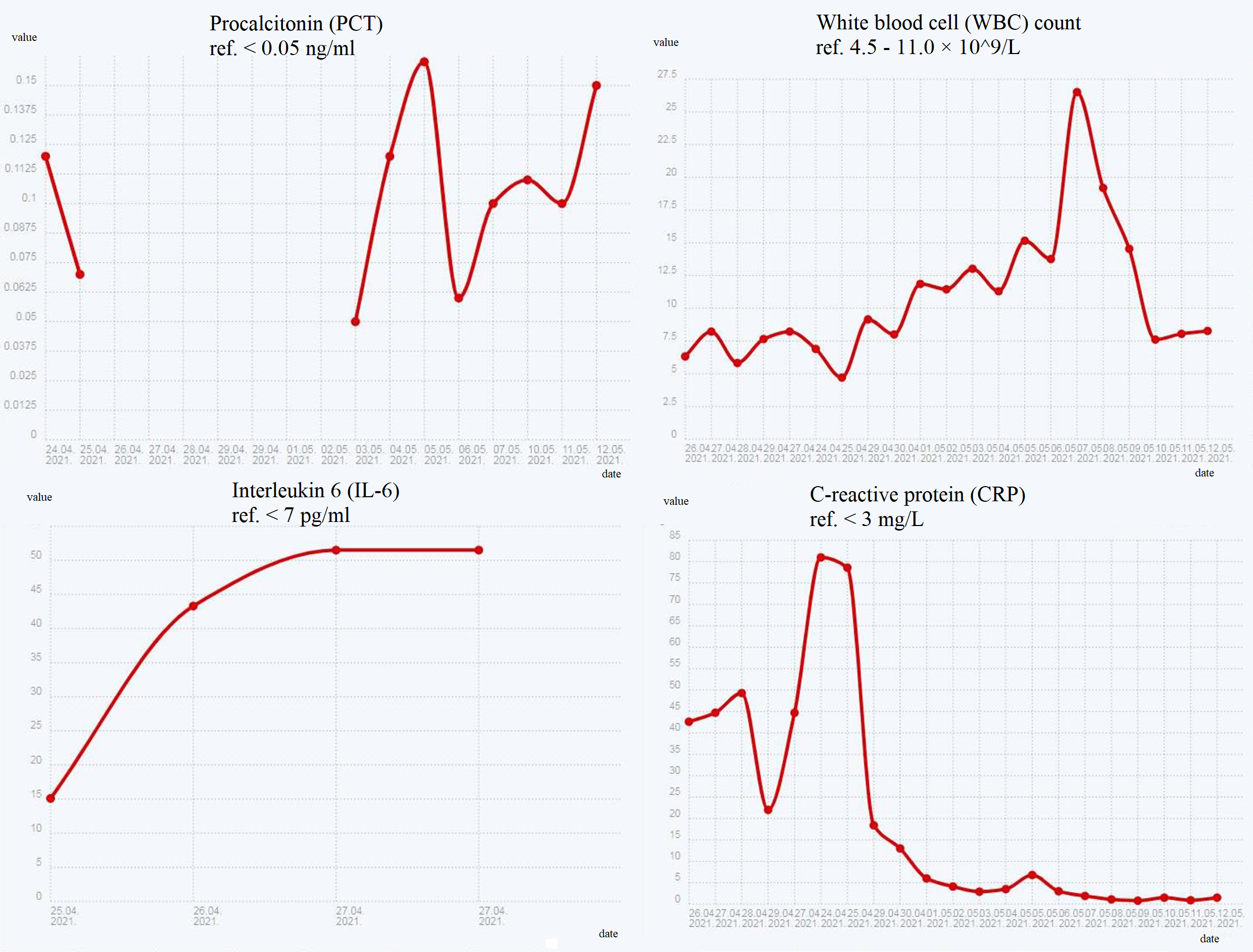

Supplement: Supplementary file 1 [file healthcare-11-01533-s001.zip › Supplementary figure S2 Dynamics of pro-inflammatory markers during the course of hospitalization.tif]
